# Collaborative Intelligent Traffic Planning in the Internet of Vehicles

**DOI:** 10.3390/s24041303

**Published:** 2024-02-18

**Authors:** Yan Zhu

**Affiliations:** School of Telecommunications Engineering, Xidian University, Xi’an 710071, China; yzhu_xidian@163.com

**Keywords:** collaborative intelligent traffic planning, internet of vehicles, vehicle on-time arrival ratio

## Abstract

With the increasing number of urban vehicles, as well as the current situation of non-intelligent traffic control systems, spatiotemporal non-uniform traffic resource occupation, and limited traffic planning and design, existing urban traffic planning methods cannot effectively solve problems such as frequent traffic congestion and uncontrollable commuting time for residents. In order to solve the above problems, this paper first constructs a multi-queue, multi-server queuing model based on the server vacation and a multi-hop cascaded queuing model from the perspective of local intersections and global commuting paths. We analyze the theoretical changes in passage delay costs at local intersections and on global commuting paths as a function of traffic flow and the random duration of traffic signals. On this basis, this article proposes a collaborative intelligent traffic planning algorithm based on artificial intelligence, which utilizes traffic sensors to dynamically perceive traffic congestion status and collaboratively plans the optimal duration of traffic signals and the optimal driving path of vehicles from both local and global perspectives, thereby maximizing the on-time arrival ratio of vehicles while ensuring the required commuting delay. The simulation results show that the proposed method can increase the on-time arrival ratio of vehicles by at least 20% compared to contrast methods while meeting the requirements relating to commuting delays. This verifies that our method can provide support for the improvement in efficiency in future Internet of vehicles.

## 1. Introduction

With the increase in urban populations, economic development, progress in new energy vehicle technology, and the reduction in vehicle costs, flexible and convenient vehicles have become an important means of transportation for people’s daily commuting, travel, and so on [1,2]. This has led to an increasing number of vehicles in cities. However, the speed and scale of urban expansion, as well as the speed of upgrading, renovating, and replanning various roads, are still far behind the growth rate of vehicle ownership [3,4]. The mismatch of multiple factors results in the existing transportation infrastructure being unable to support the peak flow of vehicles during peak hours. This phenomenon ultimately leads to a series of problems such as frequent urban traffic congestion and the inability to guarantee commuting time for residents [5,6]. The research on solving the above problems has gradually become one of the hotspots in the international transportation field.

The recent and cutting-edge studies that are directly relevant to our work involving traffic planning and the queuing model are detailed as follows. The authors in [7] build a model on a network linking a certain number of queues derived from both the taxi and urban road systems with the consideration of both the passenger and vehicle arrivals. A common multi-server M/M/c queue [8] that can account for road capacity is proposed for the urban road system, and feedback of network states is sent back to the taxi system. Nevertheless, only the road capacity is considered, not the delay, which actually determines the quality of service of the transportation systemThe above condition similarly exists in the work [9]. The paper [10] considers a vehicle routing problem with dynamic travel times due to potential traffic congestion. The developed approach mainly introduces the traffic congestion component based on queueing theory. The paper concentrates on the traffic flows on general roads but lacks an analysis of intersection conditions. [11] addresses the shortcoming that various policies are not compared in-depth. It develops a queuing model for a multi-lane highway and analyzes two policies. However, this work only considers the planning policies for highway conditions but not intersection conditions, which is similar to the research in the paper [12,13,14]. The authors in [15] present a model for the spacing distribution of queuing vehicles at a signalized junction based on random-matrix theory. This work considers the passage process in signalized junctions unilaterally but neglects the impact of global routes. Ref. [16] uses queuing theory to study the features of the vehicle arriving and the passing of the signalized intersection. It predicts the average traffic length and average waiting time at certain intersections. This work and [17,18] only involve the delay performance analysis but lack the optimal planning to improve the performance. The paper [19] proposes a computational model of drivers’ lateral control based on the queuing network cognitive architecture and the driver preview model about drivers’ lateral control activities. However, it studies how to control the vehicle to move or stop from the perspective of a driver rather than from the perspective of the commuting efficiency of traffic flows. Others focus on the resource allocation in information transmission in the Internet of vehicles but do not concentrate on the vehicle planning itself [8,20]. In summary, current works focus more on studying the traffic planning problem via the queuing theory from a non -collaborative mode in some specific scenarios but ignore the performance gain derived from globally collaborative planning.

In response to the current research status and problems of frequent urban traffic congestion, insufficient utilization of road resources, and unintelligent traffic management and control, this paper starts with the perspective of the commuting time required for residents to drive vehicles. Firstly, a multi-queue and multi-server queuing model based on the server vacation and a multi-hop queuing model based on cascaded queues are constructed. Several important factors affecting vehicle delay costs were analyzed in both intersections with traffic signals and non-intersection scenarios, such as the complex coupling relationship between traffic flow and the random duration of traffic signals. After obtaining the theoretical relationship, this paper constructs a collaborative intelligent traffic planning (CITP) method, including the architecture and algorithm for the Internet of vehicles (IoV) based on artificial intelligence. This method utilizes traffic sensors to dynamically perceive the state of the urban traffic environment in real time and collaboratively plans the optimal driving path for vehicles from the departure to the destination and the optimal duration of traffic signal variations. This maximizes the on-time arrival ratio of vehicles while ensuring the commuting time requirements of vehicles. Finally, this paper verifies the effectiveness of the proposed method by building a simulation platform for traffic planning in IoV. The simulation results show that the method proposed in this paper improves the on-time arrival ratio of vehicles by at least 20% compared to the contrast method while meeting the requirements of commuting delay, achieving the goal of improving the commuting efficiency of vehicles. The main innovation points of this paper can be summarized as follows:

1. We construct a multi-queue, multi-server queuing model based on the server vacation and a multi-hop cascaded queuing model and analyze the theoretical changes in driving delay costs at local intersections and on global commuting paths with the traffic flow and the random duration of traffic signals.
2. Based on the results of the delay analysis, we construct a collaborative intelligent traffic-planning architecture including local planning module, global planning module, and collaborative intelligent planning module, which lays the architectural foundation for the proposal of the collaborative intelligent traffic planning algorithm.
3. With the proposed architecture, we propose a CITP algorithm based on Markov decision process (MDP), which utilizes traffic sensors to dynamically perceive traffic congestion status, and collaboratively plans the optimal duration of traffic signals and the optimal driving path of vehicles from both local and global perspectives to maximize the on-time arrival ratio of vehicles.
4. We conduct numerous simulations to verify that our method, compared to contrast methods, can improve the on-time arrival ratio of vehicles by at least 20% while ensuring the commuting time requirements.

The remainder of the paper is organized as follows: we elaborate on the system model in Section 2. Next, we analyze the delay performance from local and global perspectives in Section 3. In Section 4, we propose the intelligent traffic planning architecture and the CITP algorithm. Then, we conduct numerous simulations and evaluations in Section 5. Finally, we conclude our paper in Section 6.

## 2. Preliminary Knowledge

In order to better understand the model and method proposed in this paper, we first introduce preliminary knowledge about queuing theory. A queueing process is described by a series of symbols and slashes A/B/X/Y/Z, where A denotes the interarrival-time distribution, B denotes the service-time distribution, X denotes the number of parallel servers, Y denotes the system capacity, and Z denotes the queue discipline [21]. Table 1 presents some standard symbols for these characteristics. For example, M/D/2/*∞*/FCFS indicates a queueing system with exponential interarrival times, deterministic service times, two parallel servers, infinite system capacity (i.e., no restriction on the maximum number allowed in the system), and first-come, first-served queue discipline. In many situations, only the first three symbols are used, e.g., the G/G/1 model in our paper. Typical practice is to omit the service capacity if no restriction is imposed (Y = *∞*) and to omit the queue discipline if it is first come, first served (Z = FCFS). Thus, M/D/2 would be the same as M/D/2/*∞*/FCFS. The symbols in Table 1 are, for the most part, self-explanatory. However, a few require further comment. First, it may appear strange that the symbol M is used for the exponential distribution. One might expect the use of the symbol E. However, this would be too easily confused with Ek, which is used for the Erlang distribution. Rather, M is used, standing for the Markovian or memoryless property of the exponential. Second, the symbol G represents a general probability distribution. No assumption is made as to the precise form of the distribution. Results in these cases apply to any probability distribution. Finally, the table is not complete. For example, there is no indication of a symbol to represent bulk arrivals or series queues. However, they are not related to the content of this paper and are not introduced too much.

## 3. System Model

This section constructs a multi-queue, multi-server queuing model based on the server vacation and a multi-hop queuing model for cascaded queues from both local and global perspectives and analyzes the theoretical mapping relationship between traffic flow, random traffic signal duration, and vehicle passage delay cost at intersections and non-intersection roads.

### 3.1. Multi-Queue and Multi-Server Queuing Model Based on Server Vacation

**Figure 1:**
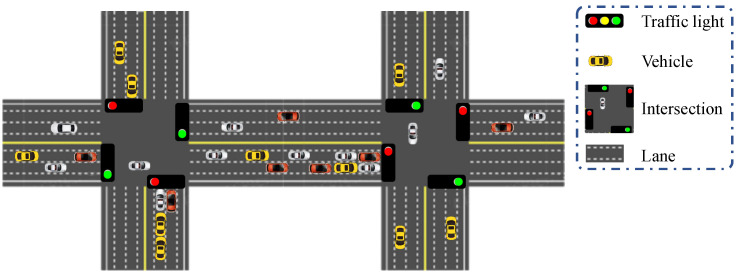
Schematic diagram of vehicle operation at traffic intersections and on general roads.

As shown in Figure 1, the multi-queue and multi-server queuing model based on a server vacation models the vehicle passage process at intersections. For queuing models, they generally consist of users, queues, and servers [22,23,24,25,26]. Users need to obtain service from servers. Before that, they should queue in line to be served one by one [21]. The delayed user passing through using this service process includes the waiting delay in the queue, the vacation delay of the server, and the service delay at the server. Reviewing the vehicle passage process at intersections, from a local perspective, there is a certain probability that vehicles need to queue up and wait for the traffic lights to change from red to green. Herein, the vehicle is modeled as a user. The intersection traffic is modeled as a server. The duration of traffic lights in red is modeled as the vacation of the server. The waiting delay depends on the moment when the vehicle arrives at the intersection and the remaining delay of the traffic light. Intersections often have multiple lanes, and newly arrived vehicles will generally enter the lane with the shortest queue length. The process of vehicles arriving at the intersection can be equivalent to the arrival process of users. The process of vehicles passing through the intersection can be equivalent to the service process of users being received from servers Moreover, the process of vehicles queuing up to wait for changes in traffic lights can be equivalent to the vacation process of servers. Multiple lanes can be modeled as a multi-queue, multi-server queuing model based on the server vacation where each lane is a single-queue, single-server queuing model.

Due to the randomness of the arrival time of vehicles at intersections and generally not following a specific distribution, the process of vehicles arriving at intersections is a general process. Similarly, the timing of traffic lights and the time for vehicles to pass through intersections generally do not follow a specific distribution. Therefore, the service process and vacation process are also generally distributed. In summary, the queuing process of vehicles on each lane can be modeled as a G/G/1 model with servers on vacation. For the delay performance of the G/G/1 model, Kramer Langenbach Belz et al. make a relatively accurate estimation [21], and the waiting delay $W_{Q}$ in the lane of intersections can be expressed as follows:

(1) $$W_{Q}=\frac{\rho /\mu \left(c_{A}^{2}+c_{S}^{2}\right)G_{KLB}}{2(1-\rho )}.$$

The corresponding correction factor $G_{KLB}$ of the G/G/1 model is

(2) $$G_{KLB}=\left\{\begin{array}{cc} \; & \exp\left(-\frac{2\left(1-\rho \right){\left(1-c_{A}^{2}\right)}^{2}}{3\rho (c_{A}^{2}+c_{S}^{2})}\right),0\leq c_{A}\leq 1,\\ \; & \exp\left(-\frac{\left(1-\rho \right)\left(c_{A}^{2}-1\right)}{c_{A}^{2}+4c_{S}^{2})}\right),c_{A}>1, \end{array}\right.$$

where ρ is the traffic intensity whose expression is $\bar{S_{t}}/\bar{A_{t}}$. $c_{A}$ and $c_{S}$ are, respectively, the covariance between the arrival time interval and service time interval whose expressions are $c_{A}=\sigma_{A}\bar{A_{t}}$ and $c_{S}=\sigma_{S}\bar{S_{t}}$. $\bar{A_{t}}$ and $\bar{S_{t}}$ represent the arrival time interval and service time interval, respectively. $\sigma_{A}$ and $\sigma_{S}$ are the standard deviations of the arrival interval and service interval, respectively.

According to the equivalent waiting time of the vacation process with a general distribution, the expression of the vacation delay $W_{V}$ in the lane of intersections is as follows:

(3) $$W_{V}=\frac{\bar{V^{2}}}{2\bar{V}},$$

where $\bar{V}$ and $\bar{V^{2}}$, respectively, represent the first and second moments of the remaining vacation time of the server corresponding to the first and second moments of the remaining second reading time of the traffic light when the vehicle arrives at the intersection [27]. The total delay cost $W_{I}$ at an intersection can be calculated as

(4) $$W_{I}=W_{Q}+W_{V}.$$

At this point, we have obtained the relationship between the traffic flow load at the intersection and the second reading time of traffic lights, as well as the delay at which vehicles pass through the intersection.

### 3.2. Multi-Hop Queueing Model Based on Cascaded Queues

As shown in Figure 1, the multi-hop queuing model based on cascaded queues models the vehicle passage process on general roads. Reviewing the vehicle passage process on general roads from a global perspective, the commuting path experienced by vehicles from the departure to the destination undoubtedly involves multiple intersections. Moreover, there are still delay costs during the driving process on general roads. The driving process of this part can be modeled as a multi-hop queuing model based on cascaded queues. Among them, each intersection and each section of roads can be regarded in terms of a single-hop queuing process. Therefore, all queuing processes from the departure to the destination together constitute the multi-hop queuing process based on cascaded queues. Similarly, the arrival process of vehicles can be modeled as a general process. Due to the determination of road speed limits, the normal driving process can be a deterministic process. The driving process of vehicles on each section of the road can be modeled as a G/D/1 model. The commuting queuing model is a multi-hop queuing model that cascades the G/G/1 model with the server vacation and the G/D/1 model. Since the G/D/1 model is a special case of the G/G/1 model, the corresponding standard deviation of service delay is 0. The total commuting delay can be written as

(5) $$W_{E}=\sum_{k=1}^{n_{t_{I}}}W_{I_{k}}+\sum_{l=1}^{n_{t_{G}}}W_{G_{l}},$$

where $n_{t_{I}}$ is the total number of intersections on the driving path. $n_{t_{G}}$ is the total number of general road parts on the driving path. $W_{I_{k}}$ is the delay cost at the *k*-th intersection. $W_{G_{l}}$ is the delay cost at the *l*-th road part that has a similar expression to $W_{I_{k}}$.

At this point, we have obtained the relationship between the traffic flow and the delay cost of vehicles passing through intersections and general roads. Furthermore, the formula for calculating the on-time arrival ratio $r_{ot}$ can be written as

(6) $$r_{ot}=\frac{\sum_{i=1}^{N_{v}}\frac{\min(0,W_{E_{i}}-W_{M_{i}})}{W_{E_{i}}-W_{M_{i}}}}{N_{v}},$$

where $N_{v}$ is the number of total vehicles, $W_{E_{i}}$ is the total commuting delay of vehicle *i*, and $W_{M_{i}}$ is the delay metric of vehicle *i* that is the allowable delay time.

## 4. Collaborative Intelligent Traffic Planning Method

In Section 2, we constructed a multi-queue, multi-server queuing model based on server vacation and a multi-hop queuing model based on cascaded queues from both local and global perspectives to characterize the commuting process of vehicles at intersections and on general roads and analyzed the theoretical mapping relationship between passage delay costs at local intersections, traffic flow, and the random duration of traffic signals. Based on this mapping relationship, we combine the local and global information, such as congestion at intersections and traffic flow on general roads (through sensor devices such as cameras and LiDAR on the road). On this basis, we propose a collaborative intelligent traffic planning method to achieve the optimal traffic planning for vehicles.

### 4.1. Collaborative Intelligent Traffic Planning Architecture

**Figure 2:**
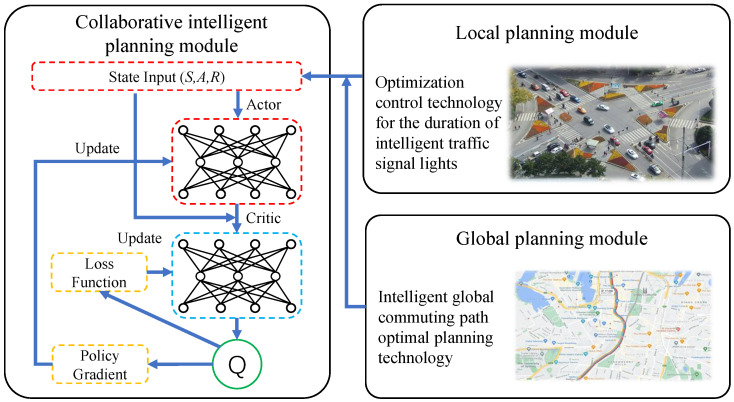
Operational framework for collaborative intelligent traffic planning methods.

This framework consists of three parts: the collaborative intelligent planning module, the local planning module, and the global planning module as shown in Figure 2. The local planning module mainly utilizes the intelligent optimization control technology for traffic light duration to achieve congestion relief at intersections. This reduces traffic congestion at intersections and local delay costs and prevents the condition that vehicles are congested in one direction with a red light while there are no vehicles in the other direction with a green light. The global planning module mainly uses intelligent commuting optimal path planning technology to achieve the best commuting path choice for vehicles from their departure to their destination. This module compensates for the lack of planning from a global perspective in the local planning module, which further improves commuting efficiency. The collaborative intelligent planning module mainly proposes a collaborative intelligent traffic planning method based on artificial intelligence to achieve collaborative linkage, perception information sharing, and learning experience interaction between local and global planning. The local and global planning modules transmit local and global information to the collaborative intelligent planning module that uses reinforcement learning methods to synchronously optimize the duration of traffic lights and the optimal commuting path.

### 4.2. Traffic Planning System Parameters

The purpose of collaborative intelligent traffic planning is to maximize the on-time arrival ratio of vehicles from the departure to the destination while being within the required commuting delay. Due to the randomness and dynamism of the number and location of vehicles in the IoV, it is difficult for traditional static optimization methods to solve this stochastic dynamic planning problem. We construct a dynamic decision-making process to describe the real-time state of the IoV, possible actions to be taken, and corresponding rewards obtained. This process can be represented by a tuple M={S,A,P,R}. S represents the set of states. A represents the set of actions. P represents the probability of state transition, and R represents the reward equation.

#### 4.2.1. State

The status of traffic planning mainly reflects the remaining delay at intersections, the number of remaining intersections, and the current traffic flow of the path. Therefore, we represent the state space at time *t* as

(7) $$s\left(t\right)=\{t_{r}\left(t\right),n_{r}\left(t\right),f_{c}\left(t\right)\}\in S,$$

where $t_{r}\left(t\right)$ is the remaining delay of the vehicle relative to its delay metric. Its expression is

(8) $$t_{r}\left(t\right)=t_{m}-t_{c}\left(t\right),$$

where $t_{m}$ is the delay metric of the vehicle. $t_{c}\left(t\right)$ is the currently elapsed delay of the vehicle at time *t*. $n_{r}\left(t\right)$ is the remaining intersection number of the vehicle from the current intersection to the destination under the current path choice. Its expression can be written as

(9) $$n_{r}\left(t\right)=n_{t}-n_{c}\left(t\right),$$

where $n_{t}$ is the total intersection number depending on the path choice. $n_{c}\left(t\right)$ is the currently elapsed intersection number of the vehicle at time *t*. $f_{c}\left(t\right)$ is the current traffic flow load at time *t* depending on $W_{A_{k}}$. We define its expression as

(10) $$f_{c}\left(t\right)=\lambda_{k}W_{I_{k}},$$

where $\lambda_{k}$ is the traffic arrival rate at the *k*-th intersection.

#### 4.2.2. Action

The action of traffic planning determines the duration of traffic signals at local intersections and the next section of the road for vehicles to travel globally. Therefore, we have

(11) $$a\left(t\right)=\{t_{l}\left(t\right),r_{x}\left(t\right)\}\in A,$$

where $t_{l}\left(t\right)$ is the duration of the signal light after taking action at time *t*. $r_{x}\left(t\right)$ is the next section of the road at time *t*.

The collaborative planning reflected in the action not only considers the duration of traffic signals at local intersections, but also takes into account the next section of the road for global vehicle travel. Through collaborative planning, the commuting efficiency of vehicles will ultimately be improved.

#### 4.2.3. Reward

It is very important for the reward of traffic planning to be well defined, which directly affects the action choice and the state variation. Our reward function is defined from the perspective of the delay satisfaction per intersection. In detail, we split the commuting delay metric by the intersection number on the path. Thus, we define the delay metric per intersection as

(12) $$t_{m_{i}}=\frac{t_{m}}{n_{t}}.$$

The reward of the *i*-th vehicle is characterized as

(13) $$r_{i}\left(t\right)=\left\{\begin{array}{cc} \frac{\beta_{i}}{n_{r}\left(t\right)}, & \mathrm{if}\;\frac{t_{r}\left(t\right)}{n_{r}\left(t\right)}\leq t_{m_{i}},\\ -\frac{\gamma_{i}}{n_{r}\left(t\right)}, & \mathrm{otherwise}, \end{array}\right.$$

where $\beta_{i}$ and $\gamma_{i}$ are positive coefficients. The form in (13) that we design is to increase the reward weights in the last few intersections because the final intersection determines whether the delay metric of the vehicle can be satisfied or not. Moreover, the total reward can be defined as

(14) $$R=\sum_{i=1}^{N_{v}}r_{i}\left(t\right),$$

where $N_{v}$ is the number of total vehicles.

From the state space we defined, it can be seen that the state of the traffic planning in the current slot is only impacted by its state in the last slot, which performs with a Markov property [28]. Therefore, this dynamic decision-making process belongs to an MDP. Because it is impossible to predict the state transition probability and reward in advance in the traffic planning environment, we propose a model-free reinforcement-learning-based traffic-planning approach to solve this MDP problem.

### 4.3. Collaborative Intelligent Traffic Planning Algorithm

Based on the constructed MDP model, we propose a CITP algorithm based on the asynchronous advantage actor–critic (A3C) to optimize the action space for improving the vehicle’s on-time arrival ratio while satisfying the commuting time requirements. The A3C is a model-free reinforcement learning architecture proposed by DeepMind and is more suitable for solving the problem with either continuous or discrete state and action spaces compared with other reinforcement learning algorithms [29]. Furthermore, it combines strategy gradient methods and value function learning methods to approximate MDP problems, especially with high-dimensional action and state spaces. The executive procedure of our CITP algorithm is detailed in Algorithm 1.

*Algorithm description:* The CITP algorithm first initializes the related parameters such as episode number, initial state, action, reward, global shared parameter vectors, thread-specific parameter vectors, upper limits of episode number, and step number. Then, the algorithm starts the repetitive optimization in each episode. The state, action, and reward are reset. The thread-specific parameters are synchronized. Each episode contains a group of steps where states are updated, actions are taken, and rewards are calculated. Then, the CITP algorithm calls the optimal path choice (OPC) algorithm as shown in Algorithm 2. The purpose is to reduce the optional range of action sets and accelerate algorithm convergence. Next, the algorithm accumulates gradients with respect to thread-specific parameter vectors and performs an asynchronous update of global shared parameter vectors. Finally, the algorithm terminates when the episode number reaches its upper limit.
**Algorithm 1:** CITP Algorithm1:**Input:**2:Initialize episode number $c_{t}$ as 1, state $t_{r}\left(c_{t}\right)\leftarrow t_{m}$, $n_{r}\left(c_{t}\right)\leftarrow m$, $f_{c}\left(c_{t}\right)\leftarrow 0$, action $t_{l}\left(c_{t}\right)\leftarrow 1$, $r_{x}\left(c_{t}\right)\leftarrow 1$, reward $r(c_{t})\leftarrow 0$, global shared parameter vectors θ and $\theta_{v}$, thread-specific parameter vectors $\theta^{\prime }$ and $\theta_{v}^{\prime }$.3:Initialize the upper limits of episode number $\hat{c_{t}}$ and step number $\hat{s_{p}}$.4:**repeat**5:   Reset state, action, and reward.6:   Step number $s_{p}\leftarrow 1$, $s\left(s_{p}\right)\leftarrow s\left(c_{t}\right)$, $a\left(s_{p}\right)\leftarrow a\left(c_{t}\right)$, gradients dθ←0 and $d\theta_{v}\leftarrow 0$.7:   Synchronize thread-specific parameters $\theta^{\prime }\leftarrow \theta$ and $\theta_{v}^{\prime }\leftarrow \theta_{v}$.8:   **repeat**9:     Call the OPC algorithm in Algorithm 2.10:     Perform $a(s_{p})$ according to policy $\pi (a\left(s_{p}\right)|s\left(s_{p}\right);\theta^{\prime })$, receive reward $r(s_{p})$ and new state $s(s_{p}+1)$.11:     $s_{p}\leftarrow s_{p}+1$.12:   **until** $s_{p}>\hat{s_{p}}$.
(15)$$R^{\prime }\leftarrow \left\{\begin{array}{cc} 0, & \mathrm{for}\mathrm{terminal}\mathrm{state}\;s\left(c_{t}\right),\\ V(s\left(c_{t}\right),\theta_{v}^{\prime }), & \mathrm{otherwise}. \end{array}\right.$$13:   **for** $i\in \{c_{t}-1,\cdots \}$ **do**14:     $R^{\prime }\leftarrow r\left(c_{t}\right)+\zeta R^{\prime }$.15:     $d\theta \leftarrow d\theta +\partial_{\theta^{\prime }}\log\pi \left(a\left(i\right)|s\left(i\right);\theta^{\prime }\right)\left(R^{\prime }-V\left(s\left(i\right);\theta_{v}^{\prime }\right)\right)$.16:     $d\theta_{v}\leftarrow d\theta_{v}+\partial_{\theta_{v}^{\prime }}{\left(R^{\prime }-V\left(s\left(i\right);\theta_{v}^{\prime }\right)\right)}^{2}$.17:   **end for**18:   Perform asynchronous update of θ using dθ and of $\theta_{v}$ using $d\theta_{v}$.19:   $c_{t}\leftarrow c_{t}+1$.20:**until**$c_{t}>\hat{c_{t}}$. =0

**Algorithm 2:** OPC Algorithm
1:Construct an undirected network topology based on vehicle delay metrics where vertices represent intersections and edges represent roads. The edge weight vector includes the delay cost of vehicle passages.2:
**repeat**
3:   Vehicles determine the road load information within two intersections by sending information to each other and updating the weights on the undirected network topology in real time.4:   If the next intersection on the shortest path meets the delay requirements for traffic flow, the entire traffic flow will be directly planned at the next intersection. If not met, plan the remaining vehicles to be at the next intersection on the shortest path, and so on until it is satisfied. If the delay metric cannot be guaranteed all the time, the algorithm terminates.5:   When a traffic accident is detected within two intersections, the shortest path involving that intersection is removed from the path, and the second-shortest path that does not include that intersection is selected for planning. After the traffic accident is over, add the relevant intersections back to the path.6:**until** The current location is the destination.


## 5. Simulation and Result Analysis

In this section, we construct a simulation environment to verify the effectiveness of the proposed model, the analysis method, and the CITP algorithm. The simulation tool involved is Matlab R2023a, which is a commercial mathematical software produced by MathWorks in the Natick, MA, USA and is used in fields such as data analysis, wireless communication, deep learning, image processing and computer vision, signal processing, quantitative finance and risk management, robotics, and control systems. The detailed simulation parameters are specified in follows.

### 5.1. Simulation Configuration

The urban transportation map originates from Xi’an, Shaanxi, China. Suppose that vehicles are randomly generated globally. The departure and destination are evenly distributed throughout the entire city. The route number depends on the all-to-all departures and destinations. The lane number per road is set to 2 for each direction. The average vehicle arrival rate, the number of intersections between the departure, and the destination are variables. From a statistical and empirical perspective, the vehicle arrival rate increases from 10 vehicles/min to 290 vehicles/min, which can reflect the impact of the vehicle arrival rate. Meanwhile, the intersection number increases from 6 to 14, which can reflect the impact of the intersection number. Moreover, the background traffic flows on the road are set as random numbers obeying uniform distribution from 55 vehicles per minute to 90 vehicles per minute. For the performance metric differentiation, the proportions of vehicles with delay metrics below 30 min, from 30 min to 50 min, and from 50 min to 70 min are set to 20%, 50%, and 30%, respectively. Signal duration and routing choice are optimization parameters obtained from our proposed CITP algorithm. Vehicles are responsible for planning and are deployed with A3C learning agents. Vehicles can connect with road sensors and other vehicles near them. More detailed parameters of our method are shown in Table 2.

### 5.2. Result Analysis

**Figure 3:**
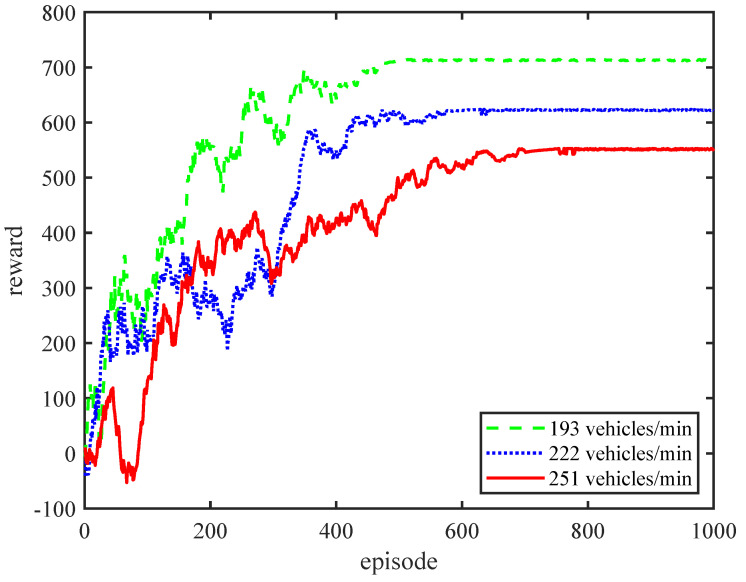
The training processes of the CITP algorithm for different vehicle arrival rates.

Figure 3 shows the trend of the reward variation with the episode during the learning process of the algorithm under different vehicle arrival rates. From the result, we can observe that the algorithm converges to its maximum reward in all cases. This verifies the convergence and effectiveness of our algorithm. The results of three scenarios show that as the vehicle arrival rate increases, the reward gradually decreases. This phenomenon indicates that as the vehicle arrival rate increases, the congestion situation in the IoV intensifies, resulting in an increase in the proportion of vehicles that cannot meet the delay metrics of each intersection. Therefore, as punishment increases, rewards correspondingly decrease. From the perspective of the convergence speed, we can see that the higher the vehicle arrival rate, the slower the convergence speed. The reason for this phenomenon is that as the vehicle arrival rate increases, the network becomes more congested, causing more vehicles to bypass paths with lighter traffic flow loads and more intersections, which leads to longer learning periods. However, the learning periods do not increase too much, which proves the good scalability of our methods.

**Figure 4:**
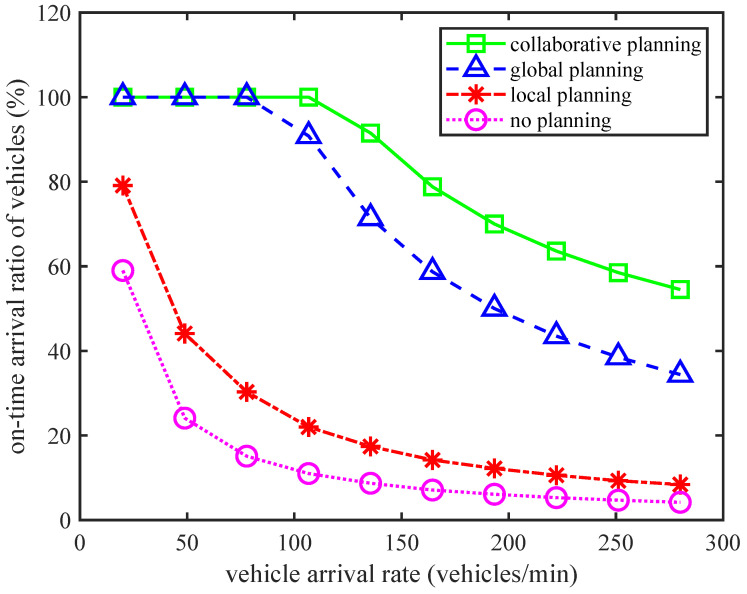
The on-time arrival ratio of vehicles vs. vehicle arrival rate for different planning strategies.

Figure 4 shows the trend of the on-time arrival ratio of vehicles with increasing vehicle arrival rate under different traffic planning strategies. From the trend of all curves, we can see that as the vehicle arrival rate increases, the on-time arrival ratio of vehicles decreases. This phenomenon indicates that when the IoV carries heavier traffic flow loads, intersection congestion and the choice of further paths will increase the commuting delay of vehicles, which will lead to an increase in the proportion of vehicles that do not satisfy delay metrics. Correspondingly, the on-time arrival ratio of vehicles will decrease. By comparing the performance of the four strategies, we can see that our proposed local and global CITP algorithm has the best performance. The followers are the only global intelligent planning strategy, the only local intelligent planning strategy, and the non-intelligent planning strategy. In addition, when the vehicle arrival rate is low, the on-time arrival ratio of vehicles based on our proposed algorithm can reach 100%. In particular, when the IoV traffic flow load tends to saturate, compared to other strategies, the on-time arrival ratio of vehicles of our algorithm is improved by at least 20%. At the same time, as the vehicle arrival rate increases, the performance of our proposed algorithm also starts to decline at the end, and the rate of the decline is the slowest. The above results consistently demonstrate the superiority of our proposed method in ensuring the on-time arrival ratio of vehicles with guaranteed delay. When vehicle arrival rates are low, the performance of the collaborative planning and global planning is the same. The reason for this result is that the light traffic load results in a very small proportion of vehicle queuing time at the intersection. At this point, the intelligent control of signal lights has the same impact on the traffic in both directions. The planning performance is mainly determined by the global planning function that the collaborative planning and global planning both have. Therefore, these two planning methods have the same performance when vehicle arrival rates are low.

**Figure 5:**
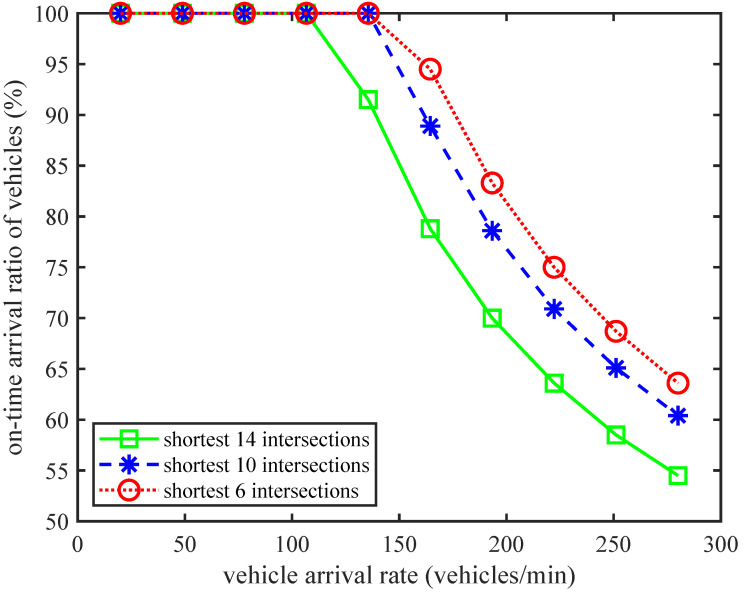
The on-time arrival ratio of vehicles vs. vehicle arrival rate for different shortest intersection numbers.

Figure 5 shows the trend of the on-time arrival ratio of vehicles with an increasing vehicle arrival rate under different shortest intersection number conditions. From the trend of all curves, we can see that as the shortest intersection number decreases, the on-time arrival ratio of vehicles increases. This phenomenon indicates that when the distance between the departure and the destination is closer, fewer intersection-passed times mean a shorter queuing delay, vacation delay, and total commuting delay. The corresponding delay cost for each hop and commuting delay cost will be reduced, which increases the on-time arrival ratio of vehicles. At the same time, we can also observe that as the shortest number of intersections decreases, the corresponding vehicle arrival rate will increase when the on-time arrival ratio of vehicles begins to decrease, and the decreasing magnitude will decrease. These phenomena indicate that when the departure and destination are closer, more vehicles can arrive on time. Therefore, the closer the commuting distance, the higher the on-time arrival ratio.

**Figure 6:**
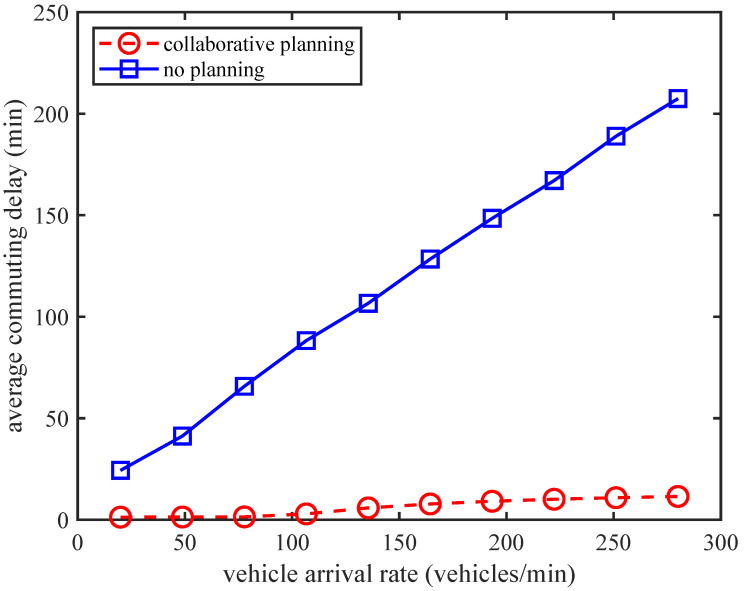
The average commuting delay vs. vehicle arrival rate for different planning strategies.

Figure 6 shows the trend of the average commuting delay of vehicles with an increasing vehicle arrival rate in different traffic planning strategies. From the trend of all curves, we can see that as the vehicle arrival rate increases, the average commuting delay also increases. This phenomenon indicates that when the IoV carries heavier traffic flow loads, the intersection congestion and the choice of further paths will increase the average commuting delay of vehicles. In addition, compared to the average commuting delay performance of the only local intelligent planning strategy and the non-intelligent planning strategy, the average commuting delay performance of the CITP algorithm, and the only global intelligent planning strategy has significantly improved, which also reflects the superiority of our proposed algorithm in terms of the average commuting delay performance. Therefore, by utilizing our proposed method, the commuting delay can be greatly reduced.

## 6. Conclusions

This paper proposes a commuting-delay-guaranteed collaborative intelligent traffic planning method to address the issues of the low on-time arrival ratio of vehicles caused by increasing scale and traffic flow load of IoV. With this method, we proposed the system architecture of the local and global intelligent traffic planning to combine two granularity planning strategies. Meanwhile, we analyzed and evaluated the local delay and global delay. Based on the results of the analysis and evaluation, we proposed a commuting-delay-guaranteed CITP algorithm based on the A3C to solve the constructed MDP problems with high-dimensional action and state spaces. Finally, we conducted extensive simulations to verify the effectiveness and superiority of our proposed method compared to contrast strategies in terms of performance such as on-time arrival ratio of vehicles and average commuting delay in different scenarios. In particular, when the IoV traffic flow load tends to saturate, compared to other strategies, the on-time arrival ratio of vehicles of our algorithm is improved by at least 20%.

## Figures and Tables

**Table 1:** Queuing notation A/B/X/Y/Z.

Characteristic	Symbol	Explanation
	M	Exponential
Interarrival-time	D	Deterministic
distribution (A)	Ek	Erlang type k(k=1,2,⋯)
Service-time	Hk	Mixture of *k* exponentials
distribution (B)	PH	Phase type
	G	General
Parallel servers (X)	1,2,⋯,∞	
System capacity (Y)	1,2,⋯,∞	
Queue discipline (Z)	FCFS	First come, first served
	LCFS	Last come, first served
	RSS	Random selection for service
	PR	Priority
	GD	General discipline

**Table 2:** Simulation Parameters.

Parameters	Values	Parameters	Values
$\bar{A_{t}}$	10∼290 vehicles/min	$n_{t}$	6,10,14
$\lambda_{k}$	55∼90 vehicles/min	$t_{m}$	30,50,70 min
$\beta_{i},\gamma_{i}$	$10(n_{t}-n_{r}\left(t\right)+1)$	learning rate	$10^{-1}$
sample time	0.02	experience horizon	256
minibatch size	32	$\hat{c_{t}}$	1000
$\hat{s_{p}}$	500		

## Data Availability

The data presented in this study are available on request from the corresponding author.

## Abbreviations

The following abbreviations are used in this manuscript:

IoV	Internet of vehicles
DRL	Deep reinforcement learning
MDP	Markov decision process
CITP	Collaborative intelligent traffic planning
A3C	Asynchronous advantage actor-critic
